# Clinical Reports in Their Relation to Homœopathy

**Published:** 1890-04

**Authors:** Clarence Willard Butler

**Affiliations:** Montclair, N. J.


### CLINICAL REPORTS IN THEIR RELATION TO HOMŒOPATHY.

CLARENCE WILLARD BUTLER, M. D., MONTCLAIR, N. J.

The consideration of clinical reports in their relation to Homœopathy, since Homœopathy is the "science of therapeutics," admits of such consideration only in respect of their relation to therapeutics, and its correlative, materia medica.

The homœopathic materia medica is woefully imperfect. No argument is needed to prove this assertion true. Our medical journals teem with pleas for a reconstructed materia medica, and our medical societies are diverted from other work to the consideration of tedious and chimerical plans for its reconstruction.

The American Institute of Homœopathy is but just delivered of the first offspring of this necessity, and, much as I dislike to cast any reflection upon her integrity, the size of the progeny proves the labor premature, while its complexion makes gravely doubtful its legitimacy.

The ideal is seldom attainable. While the ideal materia medica should contain such complete provings of every drug as will present to the student all its producible effects, from the most palpable anatomical lesion to the most trivial functional irregularity, so long as man remains unpatriotic enough to decline to kill himself slowly for the benefit of succeeding generations, our knowledge of drug action will still be, as it has heretofore been, derived from three sources, viz.: poisonings (involuntary provings), provings (voluntary poisonings), and clinical experience.

With our present lack of wisdom as to the best methods of making them available to our urgent need, all these are incomplete and unsatisfactory. The toxicological dose, acting with undue violence and rapidity, does not picture completely the functional changes which experience has taught us are the most reliable guides in prescribing; provings cannot with due regard for the health, or even the life of the prover be carried to the ultimate of drug action; clinical experience and knowledge is obtained only after so many and serious difficulties that the dangers to a pure materia medica from this source are much greater than from any other. It is, however, a perfectly legitimate source, and if its teachings are admitted to the general fund of knowledge of drug action only after repeated observations by conscientious observers; after the most rigid tests, and with the utmost precaution, it becomes a most valuable, indeed an indispensable one.

If *similia similibus curantur* formulates nature's order in curative drug effect, voices a natural law, then it is no less true that the drug which *cures* a diseased condition is capable of producing a similar condition than that the drug which produces a morbid state of animal economy is capable of *curing* a similar state; and the clinical record establishing beyond doubt the curative action of a drug is as valuable to the materia medicist and the therapeutist as is the record of drug proving. Symptoms then, subjective or objective, which have been cured by a drug may be considered as a part of that drug's action, and as such, safely embodied in our materia medica among its recorded effects.

The difficulties in determining from a clinical report whether in reality the conditions which presented themselves, and which disappeared after the exhibition of the drug were really removed by it are so many that they may well seem to preclude the possibility of gaining general knowledge from this source. The many conditions seemingly moving toward more grave and serious sicknesses, which disappear without homœopathic medication; the change in personal sanitation; in environment; in habits; the removal of aggravating accompaniments, all of which the prescriber in the exercise of his wider duties as a physician enforces, and all of which tend to make possible spontaneous recovery ; the optimistic view unconsciously taken by the always fallible mortal of his own handiwork, and indeed, many other potent factors must be weighed with due care before a positive opinion may be gained, much less a certainty of curative action be established. The danger of accepting hastily and upon too meagre evidence indications which may be unreliable must therefore be painfully evident; and, in fact, so much has been claimed and accepted by the unduly credulous as remedial effect which is based upon single experiment or partial and incomplete evidence, that it is small wonder that reports of clinical experience are regarded practically valueless as possible additions to the materia medica, by many thoughtful and careful physicians.

That from this source more errors proportionately have been foisted upon the materia medica, unintentionally to be sure, but none the less disastrously, than from any other is probably true. Nevertheless, these difficulties, various and serious as they present themselves, are not insurmountable. When repeated observations of earnest and thoughtful workers confirm the usefulness of a drug in certain and definite conditions, recognizable by well-marked signs and symptoms, then doubt gives place to certitude, and a valuable gain has been made for the uses of the healer and the restoration of the afflicted.

The introduction of new symptoms to the materia medica, then, is one sphere of the usefulness of clinical reports.

Another and perhaps a more immediately necessary one, is the confirmation of symptoms already recorded.

That many symptoms appear among the records of drug effects which are wholly the product of the too lively imagination of the prover, or of the recorder, cannot be a matter of doubt.

That many symptoms appear, the connection of which with the known action of the drug cannot be traced, but which are truly an effect of its exhibition is undoubtedly true. The personal idiosyncrasy of one prover will cause peculiar, and to us unaccountable action, which the temperament and tendencies of the majority of provers will render impossible. Such symptoms are especially valuable because, met with in the sick, they are frequently the determining symptoms for the prescription. These, too, are those symptoms which do not appear in other provings and which for that reason would be discarded by some of our most earnest and ardent materia medicists. A clinical confirmation proving their undoubted authenticity and giving them that established place in the materia medica to which they are justly entitled is a most valuable addition to the sum of our knowledge of the drug. The verification of symptoms, therefore, is a most important and practically useful function of clinical reports.

To the therapeutist those reports are most valuable which teach him from the experience of others the best *methods* of applying remedies in sicknesses.

The two questions which are least understood by the homœopathic therapeutist of to-day are the potency question and that of the repetition of doses.

To the solution of these problems, until their governing law is discovered, clinical experience affords the only guide.* It is unfortunate that controversy, often of a most bitter and uncharitable type, should have arisen over these moot questions, for they are of paramount importance, and to their satisfactory solution all earnest men should give their unbiased and honest efforts.

Since no scientific man to-day questions that all things in nature move in regular order—are governed by natural law—there can be no doubt that the time will eventually come when the law which governs here shall be known and be made practically available. And it will be when a large number of clinical reports shall have furnished sufficient data for extended comparisons that such a generalization can be made.

Until that happy time the uses of various potencies, and of the single dose, or the dose frequently or unfrequently repeated, will be especially valuable to all candid and unbiased students for comparison with their own methods in practice, and that of differing prescribers. And he who attains to the highest will not hesitate to try other methods than those he has usually employed if they promise more favorable results. Only fools discard that which they cannot understand; wise men accept the results of honest experiment, and wait for wider knowledge to solve the unknown *modus operandi.*

The selection of the remedy based upon the symptoms presenting in the case of sickness, and the symptoms recorded among the effects upon the healthy individual of the drug chosen,

* The writer is cognizant of the fact that some physicians have thought that a certain relationship existed between the doses used in the provings of the drug and that potency most useful in its exhibition in sicknesses, but since, so far as he has observed, this relationship is wholly a matter of theory, and since there seems abundant testimony that no such relationship is traceable, he has ignored the theory altogether in the above statement. To Dr. E. M. Hale's theories in posology no reference is made for the same reasons.

though a laborious task may often be accomplished, and well accomplished, by the neophyte in homœopathics; the management of the case after its exhibition frequently calls for the highest and widest knowledge of the expert in that science.

Any case, then, the history of which may cast any light upon the moot questions of homœopathic therapeutics; the question of potency; the repetition of doses; the clinical proofs of medicinal action; the solution of the often difficult question whether such action is radical and curative or only superficial or palliative; the intercurrence of anti-miasmatic remedies in the course of treatment; all of these, and, indeed, many other matters of the highest practical importance, in records of illustrative clinical cases, are in the most positive manner important and useful.

If the above propositions are true, the class of cases which should be reported, and the style and form of their presentation are not matters difficult of decision.

There is no longer need to publish cases which prove the action of the homœopathic remedy. The recorded experience of thousands of competent observers through many years has so amply established the truth of the homœopathic law that only he who is wilfully blind or hopelessly stupid may longer entertain doubt.

The labors of the clinician are for the advancement of medical knowledge, he cannot waste his time in writing for the knavish or the stupid.

It is generally useless to record failures. There is a feeling on the part of medical students that the records of failure are of the highest value. To the empirical therapeutist, he who bases his prescriptions upon the experience or theories of others, such records of failure may have value, but to the homœopathician it it will seldom occur that the record of failures is anything besides a proof of his own fallibility—a history of his own mistakes in estimating the relationship of drug to disease. Such records are valuable to the prescriber, but wholly valueless to the great body of the profession.

On the other hand, that experience which shows to the mind of the recorder undoubted cure of conditions or symptoms, new or thus newly confirmed by a given drug is of great value. Such record does not establish the right to consider these symptoms a certain and reliable indication for the drug, but it calls the attention of others to that, which, after a time, accumulated experience may confirm, and thus add to the armamentarium of the profession. And from this it follows that all cases should be reported which confirm the observations of others in clinical additions to the materia medica, or which are confirmatory of drug provings.

I think that any habitual reader of our medical literature can but regret the hasty, and too often slovenly, manner in which many valuable papers are presented; and, therefore, at the risk of seeming presumptuous in dictating to the members of a learned profession, I shall make one suggestion in respect to the style which should be employed in clinical reports.

Having chosen such cases as shall in the ways above mentioned add to the general fund of medical knowledge, present your paper in just as few words as will suffice to bring clearly in view the especial point or points which have inspired the writing. The general character and trend of the sickness having been stated, it is superfluous to give in elaborate detail the generic symptoms. The determining symptoms alone are valuable to your reader, and any elaboration of tedious detail will but obscure the central truth which you desire to promulgate or the especial fact which you wish to record. Of course, you will be accused by self-appointed critics of "prescribing for one symptom," of "ignoring the disease," etc., etc. But you cannot afford to waste your own time, or that of your thoughtful reader, that you may stop the braying of every donkey who wears a lion's skin.

Having established your position, or having completed the narration of facts, stop; and it occurs to me, Mr. Chairman, that it is fitting that I should now follow my own advice in this respect.

